# Accounting for diverse transposable element landscapes is key to developing and evaluating accurate de novo annotation strategies

**DOI:** 10.1186/s13059-023-03118-1

**Published:** 2024-01-02

**Authors:** Landen Gozashti, Hopi E. Hoekstra

**Affiliations:** Department of Organismic and Evolutionary Biology, Museum of Comparative Zoology, and Howard Hughes Medical Institute, Harvard University, Cambridge, MA 02138 USA

## Abstract

Transposable elements (TEs) are important drivers of genome evolution. Nonetheless, TE annotation remains a complex and challenging task. As more genomes from phylogenetically diverse species are published, a comprehensive pipeline for accurate annotation of diverse TEs is increasingly important. Recently, (Ou et al. Genome Biol. 20:275, 2019) developed a new comprehensive pipeline, Extensive De novo Transposable element Annotator (EDTA), and benchmarked its performance on the genomes of three species: maize, wheat, and fruit fly. Because TE landscapes can vary tremendously across species, we tested EDTA’s performance on four additional genomes with different TE landscapes: mouse, zebrafish, zebra finch, and chicken. Our analysis reveals that EDTA faces challenges with repeat classification in these genomes and underperforms overall relative to its benchmark dataset. Notably, EDTA consistently misclassifies nonLTR retrotransposons as DNA transposons, resulting in erroneous TE annotations for species with considerable repertoires of nonLTR retrotransposons. Overall, we set expectations for EDTA’s performance on genomes spanning additional diversity, urge caution when using EDTA on genomes with divergent TE repertoires from the species on which it was initially benchmarked, and hope to motivate the development of methods that are robust to both the diversity of TEs and TE landscapes observed across species.

**Supplementary Information:**

The online version contains supplementary material available at 10.1186/s13059-023-03118-1.

## Genomic diversity contributes to the burden of TE annotation

Accurate transposable element (TE) annotation represents a longstanding problem in genomics. A wealth of tools have been developed for de novo TE discovery and annotation. Nonetheless, many pipelines are specialized for specific types of TEs and/or produce highly fragmented TE libraries that require manual curation; thus a comprehensive systematic pipeline for accurate TE annotation in new genome assemblies remains elusive. In an effort to fill this gap, Ou et al. [1] benchmarked several available TE tools and used the most robust of these to develop a comprehensive pipeline for TE annotation: Extensive De novo Transposable element Annotator, EDTA.

Ou and colleagues tested EDTA on the genomes of three species — rice, maize, and fruit fly — and demonstrated its ability to produce high-quality non-redundant TE annotations for these genomes. However, TE landscapes differ drastically across eukaryotic lineages [2]. For example, while rice, maize, and fruit fly all have active DNA transposons and are dominated by LTR retrotransposons, in most mammalian genomes, DNA transposons exist only as relics of anciently active elements, and nonLTR retrotransposons (LINEs and SINEs) are the most common TE. Ou and colleagues voice awareness of such differences, but still suggest that most specialized TE tools are “agnostic to species.” Ou and colleagues also acknowledge that EDTA does not perform as well in identifying nonLTR retrotransposons as it does for other types of TEs. However, their benchmark datasets do not contain any genomes in which nonLTRs are the most common TE, limiting their ability to evaluate EDTA’s performance on such genomes. Here, we briefly evaluate EDTA’s performance on four vertebrate genomes (mouse, chicken, zebra finch, zebrafish) with good-quality TE annotations available through UCSC (used as “ground-truth” datasets) as well as rice and fruit fly (as controls).

### Benchmarking EDTA on vertebrate genomes

**Fig. 1:**
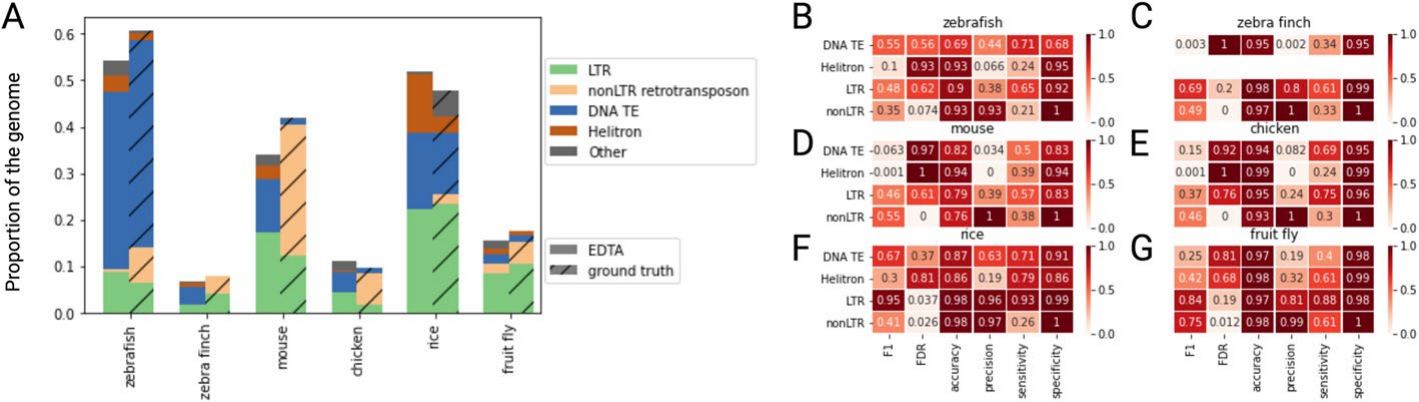
Benchmarking EDTA on vertebrate genomes.** A** Genome-wide TE content reported by EDTA relative to ground truth annotations for zebrafish, zebra finch, mouse, and chicken, as well as rice and fruit fly as controls. **B**–**G** Heat maps reporting six statistics on EDTA’s performance across different four TE types and six species

A comparative analysis of EDTA annotations and ground-truth datasets for representative vertebrate species sheds light on EDTA’s performance on genomes with TE landscapes that differ from the three benchmark species. First, although EDTA is able to recapitulate the overall repeat proportion of each genome, it struggles with TE classification (Fig. 1A). For example, EDTA falsely reports that the mouse genome is composed of ~ 10% cut-and-paste DNA transposons and < 1% nonLTR retrotransposons, when instead they account for < 3% and ~ 20% respectively (Fig. 1A). Scrutiny of differences between EDTA annotations and ground-truth annotations reveals that EDTA misclassifies at least 40% of nonLTR retrotransposons as DNA transposons in vertebrate genomes (Table 1). EDTA also misclassifies most of the remaining nonLTR elements as either helitrons or LTR elements and fails to detect a smaller subset of nonLTR elements relative to the number misclassified. Thus, although Ou and colleagues note that EDTA may face challenges with detecting nonLTR retrotransposons, misclassification seems to be the root of this problem, rather than detection. This issue could be the result of EDTA’s order of operations, since EDTA employs specialized structure-based methods for identifying LTRs, cut-and-paste DNA transposons and helitrons (and not nonLTR elements), and uses RepeatModeler to mine nonLTR retrotransposons from elements that remain unclassified thereafter [1]. Nonetheless, EDTA severely overestimates DNA transposon (and helitron) content in vertebrate genomes and consistently misclassifies nonLTR retrotransposons resulting in misleading annotations which may have affected results reported in recent publications [3–7]. It is also worth noting that EDTA seems to perform better with classifying fruit fly nonLTR elements, raising additional questions about how ascertainment bias in benchmark datasets can influence broader applicability. It is also possible that some TEs in the ground-truth datasets for the four vertebrate species we tested here are missannotated, although this seems unlikely for 10% of the genome (as in the case of the thoroughly studied mouse genome).

**Table 1:** Intersection between EDTA’s annotations and ground-truth annotations reveals EDTA’s tendency to misclassify nonLTR elements. Columns 2–7 show the percent of nonLTR elements annotated by EDTA in each of the six categories, including missed elements. The last column reports the percent of the genome occupied by nonLTR elements for each species

Species	NonLTR	Unknown	DNA	Helitron	LTR	Missed	Genome
Zebrafish	2%	0%	80%	8%	6%	4%	7%
Zebra finch	0%	0%	60%	12%	9%	19%	4%
Mouse	0%	0%	48%	10%	20%	22%	28%
Chicken	0%	0%	57%	5%	25%	14%	7%
Rice	1%	0%	36%	33%	10%	20%	2%
Fruit fly	52%	0%	16%	1%	21%	10%	5%

To quantitatively assess EDTA’s overall performance, we also employed EDTA’s benchmark companion script which compares TE annotations and calculates various benchmark statistics (F1 score, FDR, accuracy, precision, sensitivity, and specificity) (see Supplementary Materials). We find that EDTA generally displays lower F1 scores, higher FDRs, lower accuracy, lower precision, lower sensitivity, and lower specificity across all TE types in representative vertebrate genomes relative to plant genomes (i.e., rice) (Figure 1B). These discrepancies are especially pronounced for DNA transposons in genomes dominated by nonLTR elements (F1 scores < 0.2, precision < 0.1), reflecting the effects of misclassified nonLTR elements on EDTA’s performance for DNA transposons. Overall, although EDTA may be an excellent tool for TE annotation in some species, our results urge caution regarding its application to genomes with transposable element landscapes divergent from species on which it was benchmarked, such as vertebrates.

While specialized structure-based tools for specific TE types are useful for accurate annotation of full-length LTR elements and DNA TEs, they commonly struggle with TE classification, as we have seen here and previously [1, 8]. Thus, homology-based approaches remain helpful for TE classification, especially since diverse TEs can display structural similarities [9]. For genomes with divergent repeat landscapes from EDTA’s original benchmark species, Repeatmodeler represents an adequate alternative, since it uses homology to known TEs for classification, although manual curation continues to be essential for accurate full-length TE representation [10–12]. Pipelines that automate certain steps of the manual curation process (see [13]) are also promising as well as the incorporation of additional tools downstream such as those employed by EDTA.

TEs are important drivers of eukaryotic genome evolution and have contributed to innovations in adaptive evolution. However, de novo TE annotation remains a nontrivial task. As more high-quality genomes are sequenced across phylogenetically diverse species, it is paramount that we develop comprehensive TE annotation pipelines that are robust to a diversity of transposable element landscapes. Benchmarking TE annotation pipelines using a broad set of genomes is also important to fully evaluate pipeline performance and to provide prospective users with more detailed expectations for how a pipeline might apply to their respective system.

### Supplementary Information

**Additional file 1. **Downloading relevant data; Running and benchmarking EDTA

## Data Availability

The datasets supporting the conclusions of this article are available from UCSC (https://genome.ucsc.edu/) and the rice genome annotation project (http://rice.uga.edu/).
