# Supplementary material for: Accounting for diverse transposable element landscapes is key to developing and evaluating accurate de novo annotation strategies
Source: Genome Biol. 2024 Jan 2;25:4. doi: 10.1186/s13059-023-03118-1 (PMC10763064; doi:10.1186/s13059-023-03118-1)
Supplement: Supplementary file 1 — Additional file 1. Downloading relevant data; Running and benchmarking EDTA [file 13059_2023_3118_MOESM1_ESM.pdf]

Supplementary Materials

Downloading relevant data

We downloaded genomes and transposable element annotations from UCSC (https://genome.ucsc.edu/) for zebra finch (*Taeniopygia guttata*, accession: taeGut2), fruit fly (*Drosophila melanogaster,* accession: dm6), zebrafish (*Danio rerio*, accession: danRer11), mouse (*Mus musculus*, accession: mm39), and chicken (*Gallus gallus domesticus*, accession: galGal6). We retrieved the genome and transposable element annotation for rice from the rice genome annotation project (http://rice.uga.edu/).

Running and benchmarking *EDTA*

We ran *EDTA* (*--anno 1 --sensitive 1 --threads 10*, no additional filters) on each genome with no additional filtering. We ran *EDTA*'s companion benchmark script *lib-test.pl* (https://github.com/oushujun/EDTA) for each species with the curated TE annotation as the standard annotation (*-std*) and *EDTA's* annotation as the test annotation (*-tst*) for nonLTR retrotransposons (*-cat nonLTR*), LTR retrotransposons (*-cat LTR*), TIR DNA transposons (*-cat TIR*), and helitrons (*-cat Helitron*).
